# Comparison of methods to homogenize ricotta cheese samples for total solids determination

**DOI:** 10.3168/jdsc.2022-0260

**Published:** 2022-10-28

**Authors:** G. Mangione, M. Caccamo, G. Farina, G. Licitra

**Affiliations:** 1 Department Di3A, University of Catania, via Valdisavoia 5, 95123 Catania, Italy; 2 Consorzio Ricerca Filiera Lattiero-Casearia (CoRFiLaC), Regione Siciliana, 97100 Ragusa, Italy

## Abstract

Ricotta cheese is an Italian dairy product obtained by heat-coagulation of the proteins in whey, resulting from cheese production. The homogenization method applied to collect ricotta cheese samples could affect the total solids (TS) contents of the products. The aim of this study was to determine the effects of 5 different homogenization methods of the samples collection applied for the accuracy in TS determination in ricotta cheese, including un-homogenized method (UNH), un-homogenized combined with the Ultra-Turrax (IKA-Werke GmbH & Co. KG) method (UNH-UTX), spoon-homogenized method (SPN), spoon-homogenized combined with Ultra-Turrax method (SPN-UTX), and Ultra-Turrax homogenized method (UTX). The repeatability and the standard deviation of repeatability are indicators of agreement between repeated measures for TS contents. Results reported that UNH ricotta cheese samples showed large variation in TS content with values ranging from 18.31% to 25.85% and a standard deviation of repeatability higher than 1%; SPN samples showed repeatability values higher than 0.35% and standard deviation of repeatability ranged until 1.36%, suggesting large variability even in this case; the Ultra-Turrax homogenization reported repeatability values lower than 0.1% and standard deviation of repeatability lower than 0.05%, indicating that this method provides repeatable measurements that may reduce the sources of uncertainty in TS determination.

## Highlights

• The homogenization method applied to collect ricotta cheese samples affected the TS contents of the products.
• The repeatability and the standard deviation of repeatability are indicators of agreement between repeated measures for TS contents.
• Ultra-Turrax homogenization provides repeatable measurements when compared with others.
• The use of a better homogenization method results in a better estimate of the data, reducing sources of uncertainty.

## Graphical Abstract

**Figure:**
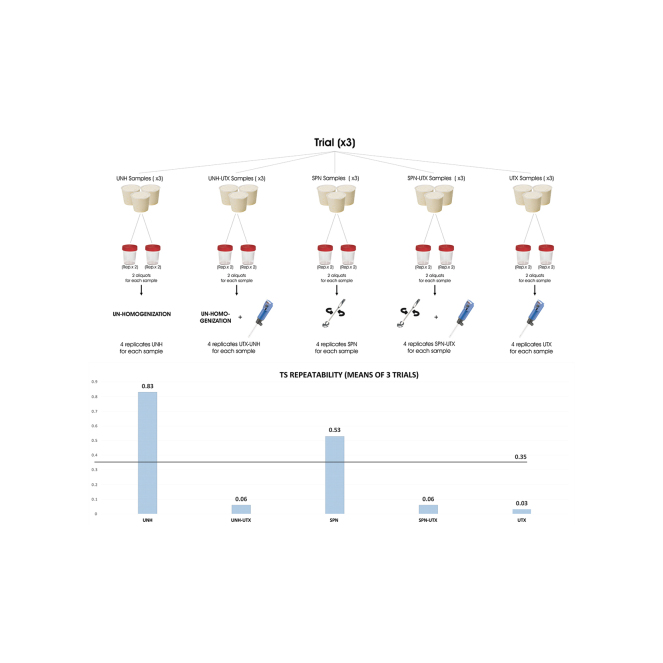
**Summary:** We evaluated the effects of 5 different homogenization methods of the samples collection applied for the accuracy in total solids (TS) determination in ricotta cheese, including un-homogenized method (UNH), un-homogenized combined with Ultra-Turrax method (UNH-UTX), spoon-homogenized method (SPN), spoon-homogenized combined with Ultra-Turrax method (SPN-UTX), and Ultra-Turrax homogenized method (UTX). Correct sample collection requires an appropriate homogenization of the product, which gives exhaustive results about the TS content. Results from UNH and SPN ricotta cheese samples showed large variation in TS content. Ultra-Turrax homogenization reported repeatable measurements that may reduce the sources of uncertainty and gives exhaustive results about the TS content.

Total solids are an important parameter for foods, including dairy products, affecting the final quality of the product along with its composition, preservation, and resistance to deterioration. The water content of food strongly influences the product's stability (Reh and Gerber, 2003). The moisture content determination is fundamental to calculate the content of the TS dairy product constituents, referred to as the DM that remains after moisture analysis.

This analytical parameter is of great economic importance to food manufacturers. Correct measurement of moisture and subsequent TS content in dairy products is important for the cheesemaker from a regulatory, economic, and stability viewpoint. Nevertheless, an accurate determination of these parameters is difficult because of the high variation from batch to batch and the long time from production.

Common procedures for sampling, sample manipulation and storage, and sample preparation could be the greatest potential source of mistakes and variation in this analysis (Bradley, 2010). Thus, adequate and repeatable methods of collection and manipulation of samples are extremely important for appropriate determination of TS in dairy products. Among these, one of the main dairy products with a high moisture level is ricotta cheese.

Ricotta cheese is a high-moisture soft dairy product not properly classified as cheese because it is produced from whey, the by-product from cheese making, and not from milk (Filippetti et al., 2007; Camerini et al., 2016; Scatassa et al., 2018). It is also allocated as a “whey cheese” within the class of dairy products reported by the Codex Alimentarius (2011) and by the Standard UNI 10978:2013 (UNI, 2013).

Typically produced in several parts of Italy, especially in the south, ricotta cheese is a traditional dairy product still produced in the artisanal way in different Italian regions, including Sicily, representing one of the main dairy products of the island.

The TS content of different fresh ricotta varieties reported in literature is highly variable between the ricotta samples, ranging from 24.28 ± 3.30, 24.86 ± 0.13, 25.84 ± 0.82, 28.92 ± 3.24, 29.41 ± 3.67, 29.97 ± 3.72, 32.13 ± 1.58, 34.49 ± 1.41, and 37.32 ± 1.97 (Mucchetti et al., 2002; Pizzillo et al., 2005; Pianaccioli et al., 2007; Filippetti et al., 2007; Mancuso et al., 2014; Tripaldi et al., 2020; Sameer et al., 2020). The main sources of variation for this determination in ricotta cheese include the time of collection from production, the correct homogenization method for the samples collection, and the sample preparation for the analysis. A precise method of sampling, indeed, includes sample preparation and homogenization techniques (ISO-IDF 707:2008; ISO-IDF, 2008). Fresh ricotta collected few minutes after the manufacture showed high percentage of moisture, due to the high presence of “scotta,” the resulting whey from ricotta production not yet drained. A correct sample collection needs an appropriate homogenization of the product, which gives exhaustive results about the TS content.

So far no studies have reported how to homogenize during the collection of ricotta cheese samples in a correct way for accurate TS determination. Therefore, the question of which sample collection procedure to use for the analysis of TS content is still valid. The purpose of this study was to determine the effects of different sample homogenization methods applied for the ricotta cheese sample collection and preparation (un-homogenized, un-homogenized combined with Ultra-Turrax, IKA-Werke GmbH & Co. KG, spoon-homogenized, spoon-homogenized combined with Ultra-Turrax, and Ultra-Turrax homogenized) on TS content determination, evaluating how the repeatability of the TS determination results was affected by the homogenization method used for the sample collection and preparation.

In this study, 5 different treatments to homogenize and prepare ricotta cheese samples were evaluated in terms of TS content determination repeatability. The experiment was replicated 3 times, and each was termed a trial. Both the sample collection and preparation and the analytical determination were performed by the same analyst.

Three ricotta cheesemaking trials were conducted, in different times, at CoRFiLaC experimental cheese plant in Ragusa (Sicily-Italy).

The ricotta cheese was produced following the traditional process, using the whey of raw sheep milk derived from Pecorino cheese making. After the curd separation, the whey was filtered and placed into a large vat, heated until 45°C was reached and salt (0.9%) was added. Subsequently, raw sheep milk (10%) was added at 50°C.

Afterward, the whey, salt, and milk mixture was heated to about 80°–85°C until the flocculated proteins rose to the surface. Once fully surfaced, ricotta cheese was manually collected and put into plastic cylindrical containers pierced to drain “scotta” whey. For the following 30 min after production, the ricotta was left to drain at room temperature, then samples were collected.

Five different homogenization method for sample collection and preparation for TS analysis determination were adopted: (1) un-homogenization (**UNH**); (2) un-homogenization–Ultra-Turrax homogenization (**UNH-UTX**); (3) spoon homogenization (**SPN**); (4) spoon homogenization–Ultra-Turrax homogenization (**SPN-UTX**); and (5) Ultra-Turrax homogenization (**UTX**).

For each method at the same time, to improve the reliability of the TS determination, 3 baskets of ricotta cheese (approximately 500 g) were randomly selected (out of 90 totally produced for each trial) and collected after 30 min of production. After the homogenization method was applied, each basket was divided into 2 aliquots per ricotta cheese sample for a total of 6 aliquots per homogenization method and a total of 30 aliquots for each trial. Each aliquot was then analyzed in duplicate for a total of 4 replicates by type of homogenization method applied.

In UNH the ricotta samples were collected without any homogenization method. For UNH-UTX each ricotta sample was first collected without any homogenization method and afterward it was homogenized with Ultra-Turrax for TS determination. Regarding the SPN method, each sample was placed into a container where the whole sample was homogenized by circular stirring for 1 to 2 min using a stainless-steel spoon. In SPN-UTX the whole ricotta samples were first homogenized by circular stirring for 1 to 2 min using a stainless-steel spoon, collected, and homogenized with Ultra-Turrax for TS determination. Finally, in UTX the whole samples were placed into a container and collocated under the Ultra-Turrax stative and homogenized using the stainless-steel dispersing elements of the instrument.

The standard procedure of determining TS content was based on weight loss. The gravimetric method (drying at 100 ± 1°C) was used according to the American Public Health Association standard (APHA, 2004) with some modifications in the analytical procedure. Approximately 2 g of ricotta was weighed into a dish and transferred to an air-drying oven at 100 ± 1°C for 24 h. Two pre-dried empty dishes were also weighed (B1, B2) on the same scale and treated in the same way of the dishes containing the samples for TS. All the samples were accurately weighed and placed in the air-drying oven at 100 ± 1°C, and after 24 h, after the samples had lost their water content, they were chilled and weighed until the difference between the 2 successive weightings did not exceed 1 mg. The weight difference measured was ascribed to the water loss; then, the TS content was determined according to the following formulas:

$$\%Moisture=\frac{\left(A-B\right)+C}{D}\times \;100,$$

% TS = 100 − % Moisture,

where A = dish and sample weight in grams; B = dish and weight of the dry sample in grams; C = average of the pre-dried empty dishes in grams (B1, B2); D = (A–E) sample weight in grams; and E = empty dish weight in grams. For all the experimental trials, each sample was analyzed in duplicate for TS determination from the same aliquot.

The statistical parameters considered to evaluate the repeatability of measures of TS using different sampling methods were the repeatability (**RT**), the standard deviation of repeatability (**St.Dev RT**), the standard deviation of the difference between the treatments methods in all samples, and the coefficient of variation of the different TS results. The RT, according to APHA (2004), is defined as the absolute difference between the results obtained in 2 different determinations, carried out simultaneously, by the same analyst and under the same conditions on an identical aliquot for the analysis. It must not exceed 0.35 g of TS per 100 g of cheese (0.35%). The St.Dev RT derived from the standard deviation of the absolute difference between the results obtained in 2 different determinations for all samples. Finally, the coefficient of variation (**CV%**) was calculated from the ratio between the mean and the standard deviation of TS values, obtained from the different methods adopted.

The ANOVA test using JMP 16 software (2022, SAS Institute Inc.) was used for the statistical analysis, considering by trial the effect of the homogenization methods (UNH; UNH-UTX; SPN; SPN-UTX; UTX) as fixed factor, whereas the variable ricotta was nested within method and the variable aliquot was nested within ricotta. Student's *t*-tests (α = 0.05) were performed to determine differences between method means when signiﬁcant effects were found.

The TS results of the UNH samples, for all trials, are shown in Table 1. The table shows the means of TS content and RT of all samples. Several differences were found in TS content in all trials with values ranging from 18.31% to 25.85%. The RT was higher than 0.60% in all 3 trials (Table 1). The St.Dev RT in all trials varied from 0.38% to 1.03%, and the CV% of all 3 trials were the highest values between the other methods of homogenization. These results suggested a large variation between the samples of the same batches. The RT tended to be better in samples with a TS content lower than 21% (0.63).

**Table 1:** Total solids content (%) of ricotta cheese samples determined using different collection and preparation methods^1^

Homogenization method	Trial 1				Trial 2				Trial 3			
	Mean (±SD)	RT (±SD)	CV%	Range	Mean (±SD)	RT (±SD)	CV%	Range	Mean (±SD)	RT (±SD)	CV%	Range
UNH	21.13 (±1.83)	1.10 (±1.03)	0.09	18.31–23.10	20.77 (±1.15)	0.63 (±0.71)	0.06	19.60–22.49	24.97 (±1.28)	0.75 (±0.38)	0.05	22.61–25.85
UNH-UTX	20.40 (±0.66)	0.06 (±0.04)	0.03	19.25–21.24	19.28 (±1.12)	0.05 (±0.06)	0.06	18.43–21.47	23.26 (±0.78)	0.07 (±0.03)	0.03	22.24–24.28
SPN	20.37 (±0.36)	0.23 (±0.32)	1.57	20.08–20.96	19.56 (±0.89)	0.19 (±0.12)	0.05	18.66–20.80	22.26 (±0.82)	1.15 (±1.36)	0.04	21.44–23.22
SPN-UTX	20.25 (±0.14)	0.04 (±0.02)	0.01	20.01–20.42	19.51 (±0.75)	0.07 (±0.04)	0.04	18.78–20.56	22.36 (±0.33)	0.09 (±0.05)	0.01	21.98–22.81
UTX	19.29 (±0.66)	0.04 (±0.02)	0.03	18.43–20.42	18.35 (±0.68)	0.02 (±0.02)	0.04	17.51–19.06	22.66 (±0.65)	0.04 (±0.04)	0.03	21.81–23.27

1 RT = repeatability. UNH = un-homogenized samples; UNH-UTX = un-homogenized–Ultra-Turrax (IKA-Werke GmbH & Co. KG) samples; SPN = spoon-homogenized samples; SPN-UTX = spoon-homogenized–Ultra-Turrax samples; UTX = Ultra-Turrax samples.

Table 1 reports the means of TS results of the ricotta cheese UNH-UTX samples. In all 3 trials, no large difference was found between the samples (RT 0.06, 0.05, and 0.07 in trials 1, 2, and 3, respectively) with a St.Dev RT lower than 0.1% (0.04, 0.06, and 0.03 in trials 1, 2, and 3, respectively), revealing a reduction of the uncertainty of the results. Moreover, the UNH -UTX samples showed low variation compared with UNH samples.

The means of TS and RT of the ricotta SPN samples, homogenized with a spoon, are reported in Table 1. The RT were lower than 0.35% in trials 1 and 2 (0.23 and 0.19, respectively) and higher than 0.35% in trial 3 (1.15).

The St.Dev RT were 0.32%, 0.12%, and 1.36% for trials 1, 2, and 3, respectively, suggesting a large variability in the TS contents of ricotta. This could be related to the low reliability of the sample homogenization method applied (with a spoon).

The TS results of ricotta cheese SPN-UTX samples, homogenized first with a spoon and afterward with Ultra-Turrax, are shown in Table 1. No out-of-range values were detected (RT higher than 0.35%), with a St.Dev RT ranging between 0.02% to 0.05% in the 3 trials.

These results could be related to the higher accuracy of the homogenization method used during the sample preparation for the analysis of the TS.

The comparison between the TS results of SPN and SPN-UTX samples showed that samples homogenized with a spoon and Ultra-Turrax had the lowest variation in TS content (CV% ranging from 0.01 to 0.04).

These results revealed better accuracy values of the TS content of ricotta cheese when homogenization with Ultra-Turrax was also adopted.

Descriptive statistics for the TS content of UTX samples, homogenized with Ultra-Turrax, are shown in Table 1. All TS values (mean and RT) showed differences lower than 0.1% with a St.Dev RT lower than 0.05% in all the samples. These represent the highest reliability, considering the low difference range between the duplicates (0.04, 0.02, and 0.04 in trials 1, 2, and 3, respectively), exhibiting that the use of Ultra-Turrax method in ricotta TS determination gave better and more accurate results that may reduce more sources of uncertainty.

Regarding the accuracy, as reported in Table 2, the statistical analysis showed that UNH samples were significantly different (*P* < 0.0001) than the other samples in all trials, attesting to the low reliability of the method in TS determination. No significant differences were found between UNH-UTX, SPN, and SPN-UTX samples in trials 1 and 2, whereas trial 3 instead UNH-UTX was significantly different from SPN and SPN-UTX samples (*P* < 0.0001).

**Table 2:** Statistical difference of the different homogenization methods for TS determination in ricotta cheese^1^

Item	Trial 1	Trial 2	Trial 3
UNH	21.13^a^	20.77^a^	24.97^a^
UNH-UTX	20.40^b^	19.28^b^	23.26^b^
SPN	20.37^b^	19.56^b^	22.26^c^
SPN-UTX	20.25^b^	19.51^b^	22.36^c^
UTX	19.29^c^	18.35^c^	22.66^bc^
*P-*value^2^	<0.0001	<0.0001	<0.0001

a–c Means within a row with different letters are significantly different (*P* < 0.001).

1 UNH = un-homogenized samples; UNH-UTX = un-homogenized samples prepared with Ultra-Turrax (IKA-Werke GmbH & Co. KG); SPN = spoon-homogenized samples; SPN-UTX = spoon-homogenized samples prepared with Ultra-Turrax; UTX = Ultra-Turrax samples.

2 *P* < 0.0001 = significant difference between the mean values.

Finally, UTX samples were significantly different (*P* < 0.0001) in trials 1 and 2 compared with the other homogenization methods, confirming that the homogenization of the whole sample with Ultra-Turrax gave a much more accurate determination of TS values in ricotta cheese.

Overall means of RT for the different samples treated with Ultra-Turrax were at 0.06 for UNH-UTX and SPN-UTX samples and 0.03 for UTX samples compared with 0.35 according to the request test method of APHA standard data.

This study contributes to clarify that a correct method of homogenization of the whole sample during the collection phase minimizes the possibility of error in TS content determination of ricotta cheese.

Results of this study indicate that the TS evaluation of ricotta cheese by Ultra-Turrax homogenization provides repeatable measurements when compared with others. The use of a better homogenization method, therefore, results in a better estimate of the data, reducing sources of uncertainty. However, further studies are needed to validate the method as a possible standard method to be applied to ricotta cheese sampling. All of these aspects require specific investigations.
